# Long-Term Performance Comparison of Bipolar Active vs. Quadripolar Passive Fixation Leads in Cardiac Resynchronisation Therapy

**DOI:** 10.3389/fcvm.2021.734666

**Published:** 2021-11-22

**Authors:** Fabian Schiedat, Harilaos Bogossian, Dominik Schöne, Assem Aweimer, Polykarpos C. Patsalis, Christoph Hanefeld, Andreas Mügge, Axel Kloppe

**Affiliations:** ^1^Department of Cardiology and Angiology at University Hospital Bergmannsheil Bochum of the Ruhr-University Bochum, Bochum, Germany; ^2^Department of Cardiology and Angiology at Marienhospital Gelsenkirchen, Gelsenkirchen, Germany; ^3^University of Witten-Herdecke, Witten, Germany; ^4^Department of Internal Medicine at Elisabeth Krankenhaus Bochum of the Ruhr University Bochum, Bochum, Germany

**Keywords:** cardiac resynchronisation therapy, active fixation, left ventricular lead, lead dislodgement, biventricular pacing

## Abstract

**Background:** Bipolar active fixation (BipolarAFL) and quadripolar passive fixation left-ventricular leads (QuadPFL) have been designed to reduce the risk of phrenic nerve stimulation (PNS), enable targeted left-ventricular pacing, and overcome problems of difficult coronary venous anatomy and lead dislodgment. This study sought to report the long-term safety and performance of a BipolarAFL, Medtronic Attain Stability 20066, compared to QuadPFL.

**Methods:** We performed a single-operator retrospective analysis of 81 patients receiving cardiac resynchronization therapy (CRT) (36 BipolarAFL, 45 QuadPFL). Immediate implant data and electrical and clinical data during follow-up (FU) were analyzed.

**Results:** BipolarAFL has been chosen in patients with significantly larger estimated vein diameter (at the lead tip: 7.2 ± 4.1 Fr vs. 4.1 ± 2.3 Fr, *p* < 0.001) without significant time difference until the final lead position was achieved (BipolarAFL: 20.9 ± 10.5 min, vs. QuadPFL: 18.9 ± 8.9 min, *p* = 0.35). At 12 month FU no difference in response rate to CRT was recorded between BipolarAFL and QuadPFL according to left ventricular end-systolic volume (61.1 vs. 60.0%, *p* = 0.82) and New York Heart Association (66.7 vs. 62.2%, *p* = 0.32). At median FU of 48 months (IQR: 44–54), no lead dislodgment occurred in both groups but a significantly higher proportion of PNS was recorded in QuadPFL (13 vs. 0%, *p* < 0.05). Electrical parameters were stable during FU in both groups without significant differences.

**Conclusion:** BipolarAFL can be implanted with ease in challenging coronary venous anatomy, shows excellent electrical performance and no difference in clinical outcome compared to QuadPFL.

## Graphical Abstract

Study design and important results. 
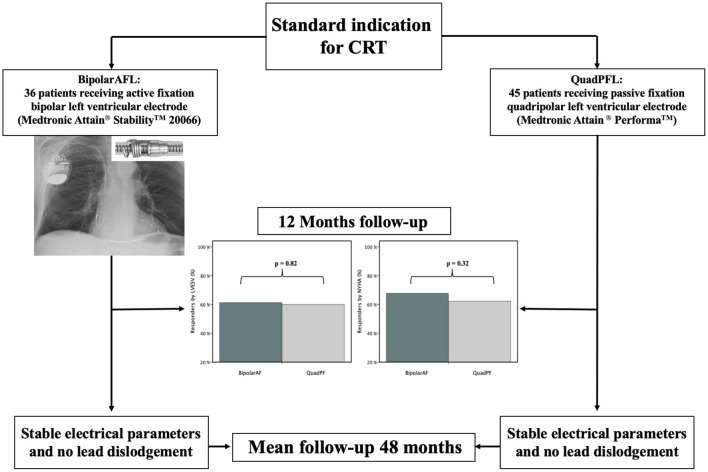

## Introduction

**Figure 1:**
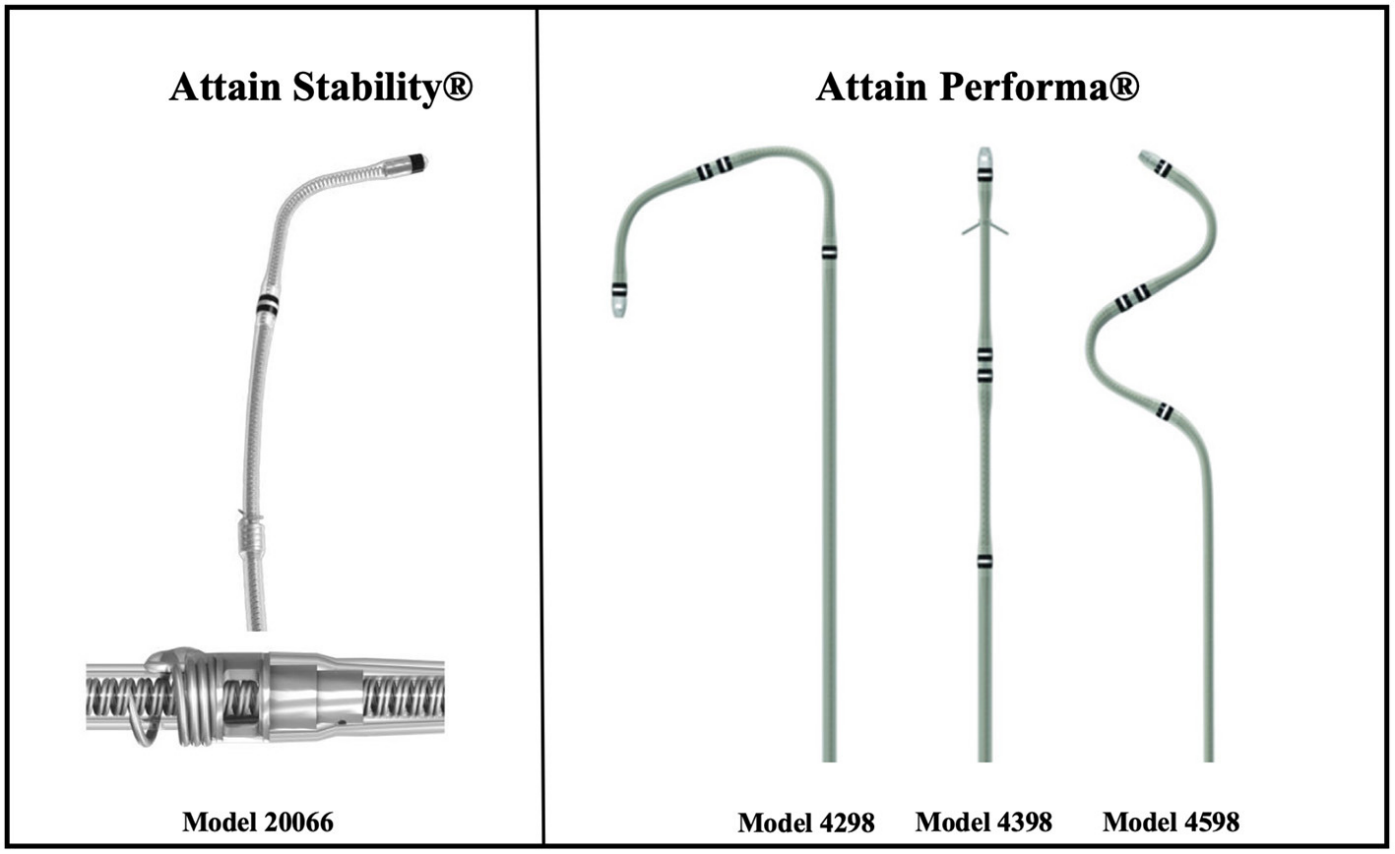
Bipolar active fixation lead (Medtronic Attain^®^ Stability^TM^ 20066) with side helix and quadripolar passive fixation leads (Medtronic Performa^®^ 4298, 4398, and 4598).

Cardiac resynchronization therapy (CRT) is a well-established therapy for patients with heart failure, reduced left ventricular-ejection fraction (LV-EF), and prolonged QRS duration. Response to CRT therapy, achieving desired LV lead placement, and LV pacing site remain a challenge until today (1, 2). With different coronary venous anatomy and size, manufacturers tried to overcome the problem of nerve stimulation (PNS), lead stability, and pacing at the desired position by manufacturing different sizes, shapes, and adding more poles. This was with limited success (3). With the Attain Stability 20066 (Medtronic, Tilburg, the Netherlands), a bipolar active fixation LV lead (BipolarAFL) has been introduced to help solve these problems. The 20066 is a 4 Fr bipolar steroid eluting lead with a small exposed side helix that is rotated clockwise into the vein wall until fixated (Figure 1). The lead has already been described in more detail elsewhere (4). First short-to-medium-term results showed good feasibility and promising clinical performance (5). Attain Performa Models 4,298, 4,398, and 4,598 (Medtronic, Tilburg, the Netherlands) is a well-established series of quadripolar passive fixation electrodes (QuadPFL) with four steroid eluting pacing electrodes (6). In this paper, we report our implant experience with BipolarAFL in comparison to QuadPFL, compare clinical outcome at 12 months between both leads and report long-term electrical results and rates of lead stability and dislodgment.

## Methods

### Study Population

We performed a retrospective analysis of patients receiving a Medtronic CRT device with either a BipolarAFL or QuadPFL. The study protocol conforms with the ethical guidelines of the 1975 Declaration of Helsinki and was approved by the local ethics committee. Patients with standard indications for CRT therapy were included in the study. Inclusion criteria were CRT implant indication in patients with impaired LV function (LV-EF ≤ 35%) and bundle branch block (BBB) according to European Society of Cardiology/European Heart Rhythm Association guidelines (7, 8). Patients were included regardless of whether they were undergoing a first-device implantation procedure or received the LV electrode as part of an upgrade from an implantable cardiac defibrillator without a prior LV lead.

### Before Implant

A total of 81 consecutive patients were included in a single center (University Hospital Bergmannsheil Bochum) in Germany. This center is specialized in CRT implantations for more than 15 years with more than 200 annual cases. It is the largest cardiovascular hospital in a city with a population of almost half a million people. Every patient received a 12-lead ECG at rest, New York Heart Association (NYHA)-class evaluation, two-dimensional transthoracic echocardiography, and medical history was collected.

### Echocardiography

Echocardiographic images were obtained by an echocardiography specialist using a transthoracic echocardiographic system (GE Vivid E9, GE Vingmed Ultrasound, Horten, Norway). The following parameters were obtained at baseline and after 12 months according to the American Society of Echocardiography guidelines: left ventricular end-systolic volume (LVESV), left ventricular end-diastolic volume (LVEDV), and LV-EF.

LV-EF was measured by the Simpson biplane method determined from the cine loops acquired in a two-dimensional model with measurement of end-diastolic and late systolic volumes in five consecutive cardiac cycles in the apical axis at focused LV views. Analysis was undertaken during postprocessing with the software EchoPAC (EchoPAC 13, GE Medical, Milwaukee, USA).

### Implant Procedure

Implantation of all three leads *via* either subclavian or cephalic vein was planned in patients with first device implantation with the right atrial lead placed in the right atrial appendage and the right ventricular lead in mid-septal position. LV lead was aimed in the basal or mid, posterolateral or lateral position after coronary venogram. The choice of LV lead was at the discretion of the implanting physician. Criteria to choose BipolarAFL included implantation from right side, aim to pace at a certain region with larger vein diameter or challenging venous anatomy according to physicians experience. All implants were performed by a single, very experienced physician (more than 12 years of implant experience at that time with an average of more than 100 annual cases). Implantation was performed under conscious sedation. For BipolarAFL, a tug test has been performed to check proper fixation. The lead position was checked and documented by fluoroscopy at 20° right anterior oblique and 20° left anterior oblique during the procedure and by x-ray the day after implantation. Vein sizes were estimated in millimeters using a catheter lab analyzation tool (Philips Xcelera, Eindhoven, the Netherlands) according to fluoroscopy at the tip, helix, and great cardiac vein. For better comparison vein size 36 mm proximal from the tip of QuadPFL has been documented as vein size at imaginary helix. To compare the vein size to lead size and have an internationally known standard size, the vein size has been converted into French (Fr). Vein angle has been measured by postprocessing as well. These measurements have been performed by three different cardiologists, blinded to the study, and the mean of the three measurements was used for further analysis.

During the procedure, all possible biventricular pacing configurations were programmed and tested for PNS with 8 V at 0.5 ms, and the threshold was tested at 0.5 ms. If pacing configurations were possible, the configuration with the longest RV to LV delay was programmed with a pacing amplitude safety margin of 1.0 V above the threshold at 0.5 ms. For QuadPFL biventricular pacing with LV pacing from a single site was programmed. The day after implant, the benefit of chosen biventricular pacing configuration was tested by echocardiography and atrioventricular optimization has been done together with an echocardiography specialist.

### Follow-Up

Device follow-up (FU) was performed 3 and 6 months after implant and every 6 months following. NYHA-class evaluation, 12-lead ECG, and two-dimensional transthoracic echocardiography assessment were performed at 12-month FU. Decrease of LVESV ≥ 15% was considered as reverse remodeling and response to CRT. Improvement of at least one NYHA class was considered as a clinical response to CRT.

### Statistical Analysis

All statistical analysis was performed using IBM SPSS Statistics version 24.0.0 on mac.

Continuous variables were stated as mean ± SD and compared with unpaired *t*-test/ANOVA for normally distributed variables and Mann–Whitney *U*-test for nonnormally distributed variables. Paired data were compared by paired *t*-test. Frequencies and percentages were reported for categorical data and compared by the chi-squared test or Fisher's exact test. Median (interquartile range) was reported for non-normally distributed data. All statistical analyses were two-sided and *p* < 0.05 was considered statistically significant.

## Results

### Demographics and Implant Procedure Bipolar Active Fixation Left Ventricular Lead

**Figure 2:**
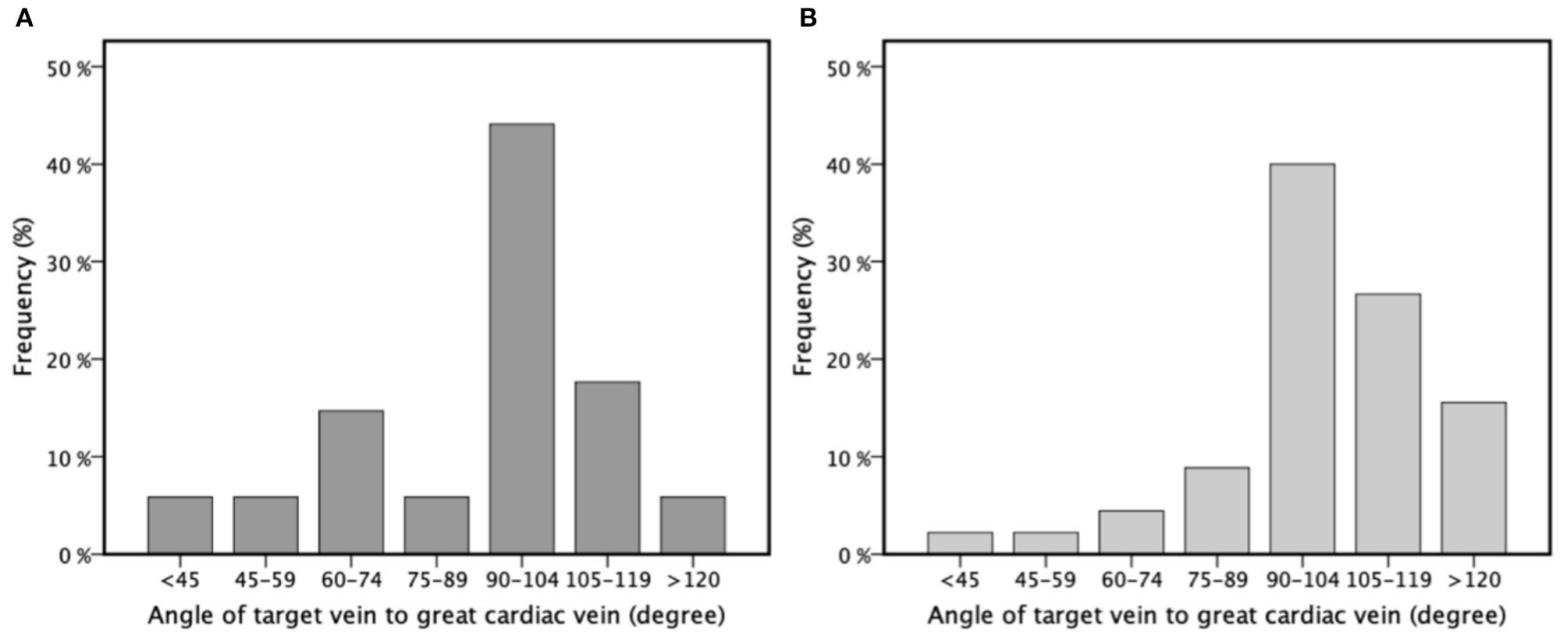
The angle of target vein to the great cardiac vein for bipolar active fixation left ventricular lead **(A)** and quadripolar passive fixation left ventricular lead **(B)** divided into different categories for better visualization presented in percentages of frequency (%) by a bar chart.

**Figure 3:**
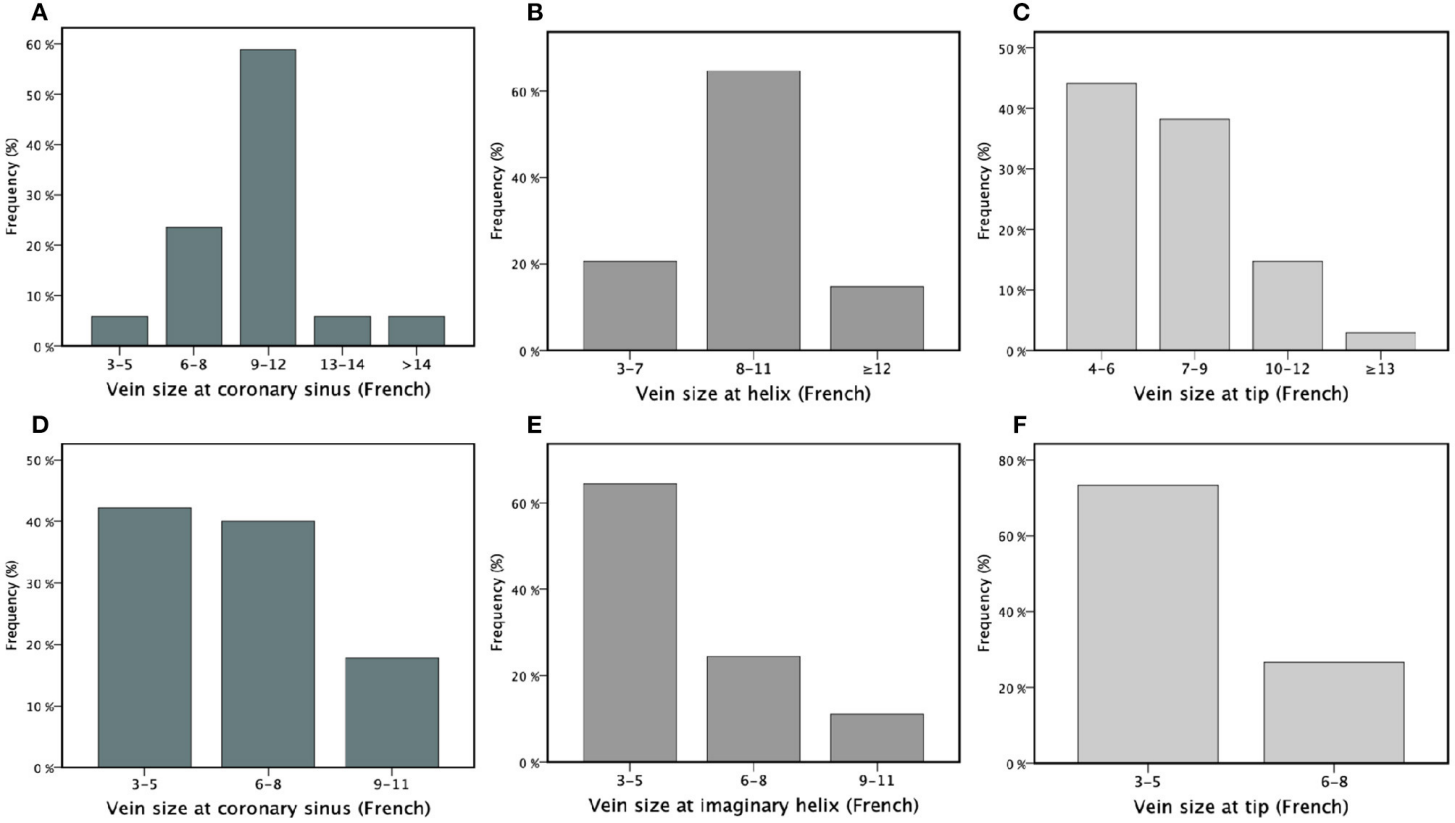
Estimated vein size for bipolar active fixation lead at coronary sinus **(A)**, the helix of the electrode **(B)** and the tip of electrode **(C)** and estimated vein size for quadripolar passive fixation group at coronary sinus **(D)**, imaginary helix position **(E)** of the electrode (36 mm from the tip) and the tip of electrode **(F)** presented in different categories of size in French (x-axis) analyzed in percentages (%) by a bar chart.

**Figure 4:**
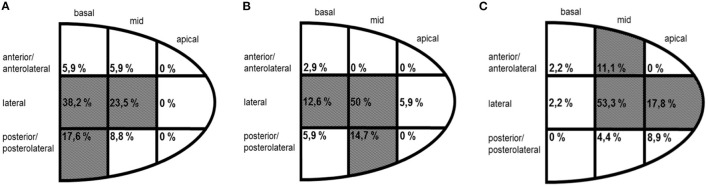
Location of the helix **(A)** and tip **(B)** of bipolar active fixation electrode and the tip of quadripolar passive fixation electrode **(C)** according to fluoroscopy shown in percentages (%) by region of the final position.

A total of 37 BipolarAFL implants were attempted between January 2014 and April 2015 with a success rate of 97.3% (*n* = 36). In one case implantation was not successful due to high thresholds at the desired position and too small vessel diameter at the tip to apply torque. The patient received a QuadPFL. The desired position was achieved in all other BipolarAFL cases and defibrillator therapy was used in 33 (91.7%) cases. Patient demographic data are summarized in Table 1. There were 26 (72.2%) first implants and 10 (27.8%) ipsilateral upgrade procedures. Right-side access was used in six (13.9%) cases. The estimated angle of the target vein was lower than 90° in 12 (33.3%) cases (Figure 2). Repositioning of BipolarAFL until the achievement of final position was necessary during 12 (33.3%) procedures. A single attempt of repositioning was necessary in nine (25%) cases, two attempts in one (2.8%), and three attempts in two (5.6%) cases. Meantime to access coronary sinus was 6.6 ± 4.3 and 20.9 ± 10.5 min until the lead was fixated at the final position. Estimated vein size at final helix position was larger than 7 Fr in 28 (78%) cases (Figure 3). The final position of the helix and tip is illustrated in Figure 4. There were no early dislodgements.

**Table 1:** Baseline data.

	**Bipolar active fixation lead** **(***n*** = 36)**	**Quadripolar passive fixation lead** **(***n*** = 45)**	***P*** **-value**
Age at implant (years)	71.8 ± 9.6	72 ± 7.6	0.71
Sex, male, *n* (%)	27 (75%)	34 (75.6%)	0.22
BMI (kg/m^2^)	29.1 ± 3.9	27.1 ± 4.2	0.16
BSA	2.0 ± 0.2	2.0 ± 0.2	0.41
Ischemic cardiomyopathy, *n* (%)	22 (61.1%)	25 (55.6%)	0.36
Myocardial infarction, *n* (%)	14 (28.8%)	21 (46.7%)	0.43
Hypertension, *n* (%)	26 (72.2%)	33 (73.3%)	0.25
Diabetes, *n* (%)	14 (38.9%)	17 (37.8%)	0.87
Chronic kidney disease, *n* (%)	13 (36.1%)	16 (28.9%)	0.09
History of stroke/TIA, *n* (%)	4 (11.1%)	6 (13.3%)	0.33
Atrial fibrillation, *n* (%)	15 (41.9%)	23 (51.1%)	0.77
Beta-Blocker, *n* (%)	35 (97.2%)	43 (95.6%)	0.75
ACE-Inhibitor/ARBs	34 (94.4%)	43 (95.6%)	0.82
MRA	32 (88.9%)	41 (91.1%)	0.72
NYHA class	2.6 ± 0.8	2.8 ± 0.6	0.21
Center bundle branch block, *n* (%)	35 (97.2%)	41 (91.1%)	0.30
QRS duration (ms)	166.4 ± 38.2	170.8 ± 26.1	0.38
Center ventricular ejection fraction (%)	29.6 ± 10.2	28.3 ± 8.3	0.54
Center ventricular end-systolic volume (ml)	120 ± 36	123.6 ± 29.2	0.15
Center ventricular end-diastolic volume (ml)	165.7 ± 54.4	168.1 ± 58.6	0.21
CRT-D implant, *n* (%)	33 (91.7%)	40 (88.8%)	0.77
Device upgrade, *n* (%)	10 (27.8%)	11 (24.4%)	0.61

### Implant Procedure Quadripolar Passive Fixation Left Ventricular Lead and Comparison to Bipolar Active Fixation Lead

A total of 46 QuadPFL implants were attempted between January 2014 and April 2015 with a success rate of 98%. In one case, the diameter of the target vein was too small to achieve wedge position and a standard bipolar lead was implanted instead. The desired position was achieved in all other patients with the final position of tip illustrated in Figure 4. Right-side access was used in three (4.4%) cases with no significant difference compared to BipolarAFL (*p* = 0.15). The estimated angle of the target vein was lower than 90° in eight (17.8%) cases (Figure 2), being significantly less than in BipolarAFL (*p* < 0.05). Repositioning of QuadPFL until achievement of final position was necessary during 10 (22.2%) procedures, not being significantly different to BipolarAFL (22.2 vs. 33.3%, *p* = 0.23). Meantime to access coronary sinus (6.6 ± 3.4 min) was not significantly different compared to BipolarAFL (6.2 vs. 6.6 min, *p* = 0.78) and neither was time until the achievement of final position (18.9 ± 8.9 min) compared to BipolarAFL (18.9 vs. 20.9 min, *p* = 0.35). Estimated vein size at imaginary helix position (36 mm from the tip of QuadPFL) was significantly smaller compared to estimated vein size at the helix in BipolarAFL (5.8 ± 3.6 Fr vs. 8.8 ± 3.4 Fr, *p* < 0.01) and at the tip compared to BipolarAFL (4.1 ± 2.3 Fr vs. 7.2 ± 4.1 Fr, *p* < 0.001; Figure 3). Tip of QuadPFL was compared to BipolarAFL significantly more often placed in a more anterior (*n* = 6, 13.3% vs. *n* = 1, 2.9%, *p* < 0.05) and apical (*n* = 12, 26.7% vs. *n* = 2, 5.9%, *p* < 0.05) position (Figure 4).

### Clinical Follow-Up

All patients completed 12 months FU. Clinical FU data are summarized in Table 2. Heart failure-associated hospitalizations occurred in two BipolarAFL (5.7%) and two QuadPFL (4.6%) patients and were not significantly different (*p* = 0.81).

**Table 2:** Clinical data during follow-up (baseline and 12-month follow-up).

	**Bipolar active fixation lead** **(***n*** = 36)**	**Quadripolar passive fixation lead** **(***n*** = 45)**	***p*** **value**
Delta QRSd (ms)	−20.8 ± 8.5	−21.3 ± 8.2	0.68
Delta LVESV (ml)	−22.2 ± 26.2	−29.6 ± 31.4	0.48
Delta LVESV (%)	−20.1 ± 20.8	−21.8 ± 22.0	0.73
Delta LV-EF absolute change (%)	+10.1 ± 7.9	+8.1 ± 8.2	0.89
Delta LV-EF relative change (%)	+29.4 ± 42.4	+24.7 ± 42.4	0.81
Delta NYHA class	−1.2 ± 1.1	−1.0 ± 1.0	0.42
Responder according to LVESV, *n* (%)	22 (61.1 %)	27 (60 %)	0.82
Responder according to NYHA, *n* (%)	24 (66.7%)	28 (62.2%)	0.32

**Figure 5:**
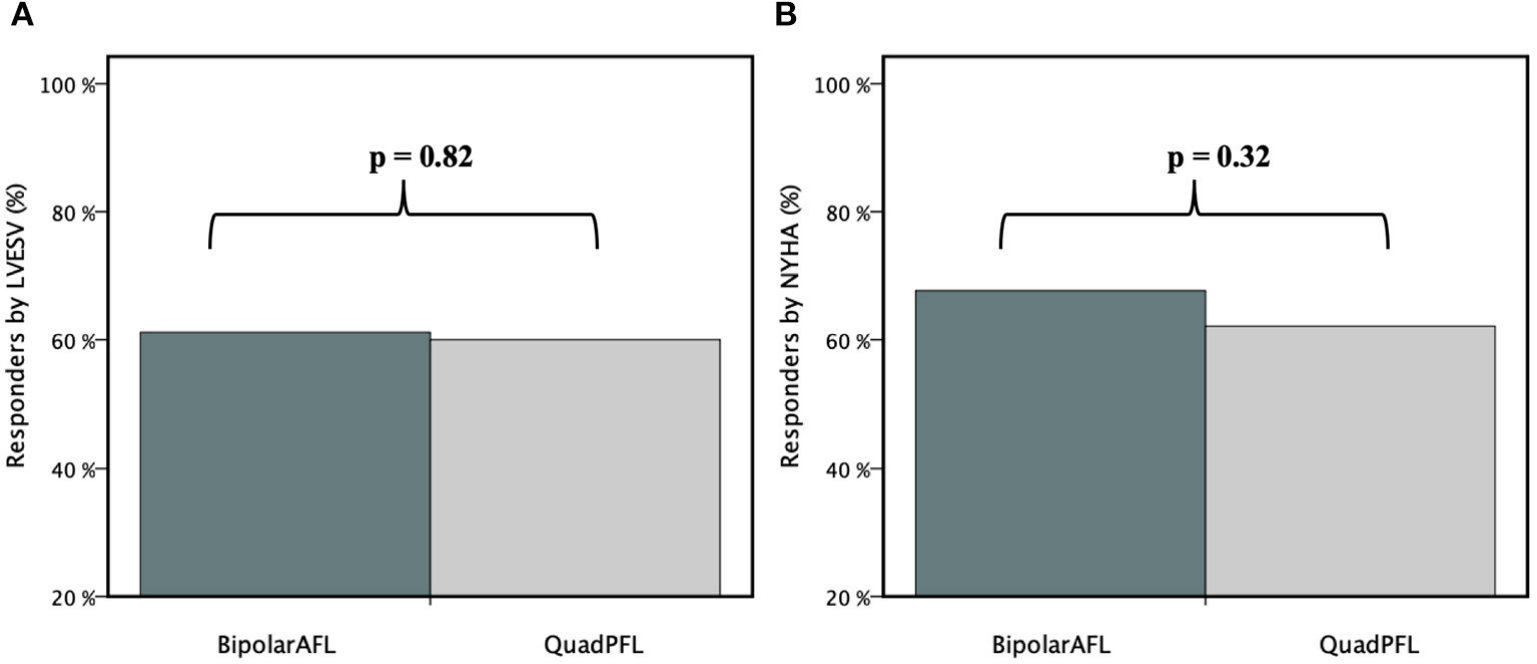
Percentage of responders (%) according to LVESV **(A)** and NYHA **(B)** compared between bipolar active fixation (BipolarAFL) and quadripolar passive fixation (QuadPFL) group by bar graph.

During 12 months FU reverse remodeling in terms of LVESV reduction was significant compared to baseline for both groups (BipolarAFL: −22.2 ± 26.2 ml, *p* < 0.001; QuadPFL: −29.6 ± 31.4, *p* < 0.001) but not significantly different between the groups (*p* = 0.48). Absolute LV-EF improvement was significant in both groups compared to baseline (BipolarAFL: +10.1 ± 7.9%, *p* < 0.001; QuadPFL: +8.1 ± 8.2, *p* < 0.001) but not significantly different between both groups (*p* = 0.89). There was no difference in response rate according to LVESV with 61.1% response rate in BipolarAFL and 60% in QuadPFL (*p* = 0.82) (Figure 5).

Improvement of NYHA class was significant for both groups compared to baseline (BipolarAFL: −1.2 ± 1.1 NYHA class, *p* < 0.01; QuadPFL: −1.0 ± 1.0 NYHA class, *p* < 0.01), but was not significantly different between both (*p* = 0.42). Response rate according to NYHA was not different with 66.7% response rate in BipolarAFL and 62.2% in QuadPFL (*p* = 0.32) (Figure 5). Shortening of QRS duration was significant in both groups during FU (BipolarAFL: −20.8 ± 8.5 ms, *p* < 0.01; QuadPFL: −21.3 ± 8.2 ms, *p* < 0.01), but was not significantly different between both groups (*p* = 0.68). There has not been a significant change of medication during clinical FU.

### Electrical Performance During Follow-Up in BipolarAFL

The median FU time was 48 months (IQR: 44–54 months). A comparison of x-ray after implant and at 12 months showed no movement of electrode or dislodgement in BipolarAFL. During further FU there was no dislodgement either. The final bipolar pacing capture thresholds (PCT) at 0.5 ms were 1.2 ± 0.6 V at implant, 1.2 ± 0.8 V at 3 months, 1.0 ± 0.6 V at 6 months, 1.0 ± 0.6 V at 12 months, 1.0 ± 0.5 V at 24 months, 1.0 ± 0.6 V at 36 months, and 1.1 ± 0.5 V at 48 months. There were no significant changes during the time (implant vs. 48 months *p* = 0.41). Bipolar pacing impedance during FU were 572 ±149 Ohms at implant, 542 ± 157 Ohms at 3 months, 512 ± 109 Ohms at 6 months, 508 ± 159 Ohms at 12 months, 538 ± 99 Ohms at 24 months, 535 ± 121 Ohms at 36 months, and 514 ± 141 Ohms at 48 months. There were no significant changes during the time (implant vs. 48 months *p* = 0.18). Sensing was good at implant 12.4 ± 6.4 mV and stable during FU with 12.5 ± 5.9 mV at 3 months, 12.4 ± 6.0 mV at 6 months, 12.5 ± 5.6 mV at 12 months, 12.5 ± 5.5 mV at 24 months, 12.4 ± 5.5 mV at 36 months, and 12.5 ± 5.4 mV at 48 months. There were no significant changes during the time (implant vs. 48 months *p* = 0.78). PNS did not occur at 8.0 V in chosen pacing configuration during FU. The mean of biventricular pacing was 99.2 ± 3.5% during the observational period.

### Comparison of BipolarAFL and QuadPFL Long-Term Electrical Performance

In QuadPFL there were no lead dislodgments during FU either. At desired pacing area, final programmed PCTs at 0.5 ms were stable during FU (1.4 ± 0.8 V at implant vs. 1.2 ± 0.7 V at 48 months, *p* = 0.34) and not significantly different in average during total FU time compared to BipolarAFL (QuadPFL: 1.3 ± 0.8 V vs. BipolarAFL: 1.1 ± 0.6 V, *p* = 0.23). Pacing impedance was stable in QuadPFL during FU (610 ± 199 Ohms at implant vs. 601 ± 205 Ohms at 48 months FU, *p* = 0.51) and not significantly different in average during FU compared to BipolarAFL (QuadPFL: 603 ± 200 Ohms vs. 531 ± 139 Ohms, *p* = 0.34). PNS at 8 V and even 5 V occurred during FU in six (13%) patients but was resolved with a change of bipolar pacing configuration. This was significantly more often compared to BipolarAFL (*p* < 0.05). The mean of biventricular pacing was 98.9 ± 2.8% during the observational period. This was not significantly different compared to BipolarAFL (*p* = 0.53).

## Discussion

The development of quadripolar leads and improved lead design has helped to reduce problems of PNS, high thresholds, and lead stability (9, 10). With the possibility of pacing at a desired area clinical outcome, the rate of hospitalization and mortality has improved as well (11, 12). Attain Performa is a well-established quadripolar lead for which these benefits have been described (13). A problem that remained and evolved to be the main problem in LV lead implant failure, is difficult coronary venous anatomy as reported in a meta-analysis by Gamble et al. (3). Our data suggest that BipolarAFL is helpful in these cases with difficulties like implantation from the right side, large coronary veins and target veins with a less steep angle, where achieving a stable wedge position with QuadPFL could be difficult. Implantation of BipolarAFL was done with ease, repositioning was possible if necessary and procedure times were comparable to QuadPFL. This is in line with data reported by Ziacchi et al. (5). A very small vein diameter however can make it difficult to screw the side helix into the vein wall as reported by us and Johar and Luqman (14). This, however, seems to be the only anatomy in which BipolarAFL is not helpful.

In this study, there were no lead dislodgments during FU in both groups. For passive fixation, quadripolar LV electrodes Erath et al. reported significantly lower rates of dislodgment requiring replacement in a meta-analysis compared to passive fixation bipolar leads, with rates however still ranging between 1 and 9% (10, 15, 16). For 20066 BipolarAFL, no cases of dislodgement after discharge have been reported so far and early dislodgment can be prevented by performing a push-test (5).

A big advantage of quadripolar leads is pacing at a mid/basal part of the vein with the tip being wedged distally. As illustrated in Figure 4, we were able to place the tip of BipolarAFL in a mid/basal part for most cases and less often in an apical and anterior position compared to QuadPFL. This is important as Kutyifa et al. demonstrated higher mortality in apical and anterior placed leads (17). Active fixation leads can be an advantage compared to passive fixation leads where insufficient wall contact of pacing poles or high PCT can prevent pacing at the desired area and therefore lead to pacing in more apical or anterior areas.

To determine prognosis, LV reverse remodeling is probably the most important marker (18). Reverse remodeling and response to CRT according to LVESV in our study conforms with larger trials with quadripolar electrodes (19). Clinical data have been collected at 12 months FU as reverse remodeling continues in most patients for 6–12 months and not further afterward (20). Electrical performance of BipolarAFL was good and stable in this long-term FU with chronically low PCTs and in line with previous reported short-to-medium-term FU data (4, 5, 14). The main difference to these studies is the longer electrical FU, which has not been reported before. PNS did occur more frequently in QuadPFL but with the change of bipolar pacing configuration, this could be resolved. With quadripolar leads, this seems to be more of an issue if modern technology like multisite pacing is desired. Extraction was not necessary during our FU for either type of lead. But even for BipolarAS, cases of easy extractability have been reported (21, 22).

## Limitations

The limitation of our study was the single-center, single-operator, non-randomized, and retrospective study design with a limited sample size. Therefore, proper analysis of lead dislodgment is limited. CRT implants after April 2015 have not been included as the goal was to have comparable groups in the long-term with similar FU duration.

As with all studies involving echocardiography, intra- and interobserver variability is a known issue. As the choice which led to implant was made at implanting physicians' discretion, it remains unclear whether passive fixation leads would have shown high stability in difficult coronary venous anatomy as well. Reported data suggest otherwise, with lead dislodgment rates between 1 and 9% for quadripolar passive fixations leads, but this remains uncertain for our cohort (15). Confounding is an issue as the lead has been chosen at the operator's discretion according to venous anatomy.

By now a quadripolar active fixation electrode (Metronic Attain Stability Quadripolar 4798) has been introduced and showed promising results in initial reports and short-term FU (23). A combination of active fixation, quadripolar lead design, and modern pacing technologies, such as multisite pacing, should be examined as they could lead to improvement in LV reverse remodeling and CRT response in a long-term FU. This technology however has not been available at the time of initiation of our study. Additionally, technologies such as His-bundle pacing or left-bundle branch pacing are other modern alternatives in selected patients where implantation of LV lead is difficult.

## Conclusion

Bipolar active fixation lead (Medtronic Attain Stability 20066) is a safe and easy implantable LV lead, even in situations with a high risk of lead dislodgement (implantation from the right side, large coronary vein diameter, or less steep target vein angle). It was not associated with the measurable difference in clinical outcome compared to quadripolar passive fixation leads. During this long-term FU reported with a median of 48 months, BipolarAFL enabled pacing at the desired area with no dislodgement, was stable with excellent electrical parameters, and showed a low incidence of PNS. Further prospective, randomized studies with a larger cohort and combination with modern pacing options would be interesting.

## Data Availability Statement

The raw data supporting the conclusions of this article will be made available by the authors, without undue reservation.

## Ethics Statement

The studies involving human participants were reviewed and approved by Ethics committee of the Ruhr University Bochum. The patients/participants provided their written informed consent to participate in this study.

## Author Contributions

FS, AM, and AK: design of study. FS, HB, DS, AA, PP, and CH: data acquisition. FS, HB, DS, and CH: data analysis. CH, AM, and AK: data interpretation. FS and HB: writing manuscript. AM and AK: revision of manuscript. All authors contributed to the article and approved the submitted version.

## Conflict of Interest

AK received speakers honoraria from Medtronic. The remaining authors declare that the research was conducted in the absence of any commercial or financial relationships that could be construed as a potential conflict of interest.

## Publisher's Note

All claims expressed in this article are solely those of the authors and do not necessarily represent those of their affiliated organizations, or those of the publisher, the editors and the reviewers. Any product that may be evaluated in this article, or claim that may be made by its manufacturer, is not guaranteed or endorsed by the publisher.

## Abbreviations

BipolarAFL: Bipolar active fixation left ventricular lead
CRT: Cardiac resynchronization therapy
Fr: French
FU: Follow-up
IQR: Interquartile range
LV: Left ventricle
LV-EF: Left ventricular ejection fraction
LVEDV: Left ventricular end-diastolic volume
LVESV: Left ventricular end-systolic volume
NYHA: New York Heart Association
PCT: Pacing capture threshold
PNS: Phrenic nerve stimulation
QuadPFL: Quadripolar passive fixation left ventricular lead.

## References

[1] Sieniewicz BJ Gould J Porter B Sidhu BS Teall T Webb J. Understanding non-response to cardiac resynchronisation therapy: common problems and potential solutions. Heart Fail Rev. (2019) 24:41–54. 10.1007/s10741-018-9734-8 30143910 PMC6313376

[2] Markewitz A Bundesfachgruppe Herzschrittmacher und D. [Annual report 2017 of the German pacemaker- and defibrillator register - part 2: implantable cardioverter defibrillators (ICD): working group on cardiac pacemaker and implantable cardioverter-defibrillators at the IQTIG - institute of quality assurance and transparency in healthcare]. Herzschrittmacherther Elektrophysiol. (2019) 30:389–403. 10.1007/s00399-019-00648-9 31705261

[3] Gamble JHP Herring N Ginks M Rajappan K Bashir Y Betts TR. Procedural success of left ventricular lead placement for cardiac resynchronization therapy: a meta-analysis. JACC Clin Electrophysiol. (2016) 2:69–77. 10.1016/j.jacep.2015.08.009 29766856

[4] Yee R Gadler F Hussin A Bin Omar R Khaykin Y Verma A. Novel active fixation mechanism permits precise placement of a left ventricular lead: early results from a multicenter clinical study. Heart Rhythm. (2014) 11:1150–5. 10.1016/j.hrthm.2014.04.020 24801899

[5] Ziacchi M Giannola G Lunati M Infusino T Luzzi G Rordorf R. Bipolar active fixation left ventricular lead or quadripolar passive fixation lead? An Italian multicenter experience. J Cardiovasc Med. (2019) 20:192–200. 10.2459/JCM.0000000000000778 30762662

[6] Lin AC Biffi M Exner DV Johnson WB Gras D Hussin A. Long-term electrical performance of attain performa quadripolar left ventricular leads with all steroid-eluting electrodes: results from a large worldwide clinical trial. Pacing Clin Electrophysiol. (2018) 41:920–6. 10.1111/pace.13389 29808920

[7] Brignole M Auricchio A Baron-Esquivias G Bordachar P Boriani G Breithardt OA. 2013 ESC guidelines on cardiac pacing and cardiac resynchronization therapy: the task force on cardiac pacing and resynchronization therapy of the European society of cardiology (ESC). Developed in collaboration with the European heart rhythm association (EHRA). Eur Heart J. (2013) 34:2281–329. 10.1093/eurheartj/eht150 23801822

[8] Ponikowski P Voors AA Anker SD Bueno H Cleland JGF Coats AJS. 2016 ESC Guidelines for the diagnosis treatment of acute chronic heart failure: the task force for the diagnosis treatment of acute chronic heart failure of the European society of cardiology (ESC) developed with the special contribution of the heart failure association (HFA) of the ESC. Eur Heart J. (2016) 37:2129–200. 10.1093/eurheartj/ehw128 27206819

[9] Ziacchi M Zucchelli G Ricciardi D Morani G De Ruvo E Calzolari V. Performance and clinical comparison between left ventricular quadripolar and bipolar leads in cardiac resynchronization therapy: observational research. Indian Heart J. (2018) 70:864–71. 10.1016/j.ihj.2018.05.007 30580858 PMC6306340

[10] Boriani G Connors S Kalarus Z Lemke B Mullens W Osca Asensi J. Cardiac resynchronization therapy with a quadripolar electrode lead decreases complications at 6 months: results of the MORE-CRT randomized trial. JACC Clin Electrophysiol. (2016) 2:212–20. 10.1016/j.jacep.2015.10.004 29766873

[11] Leyva F Zegard A Qiu T Acquaye E Ferrante G Walton J. Cardiac resynchronization therapy using quadripolar versus non-quadripolar left ventricular leads programmed to biventricular pacing with single-site left ventricular pacing: impact on survival and heart failure hospitalization. J Am Heart Assoc. (2017) 6:ee007026. 10.1161/JAHA.117.007026 29042422 PMC5721885

[12] Schiedat F Schone D Aweimer A Bosche L Ewers A Gotzmann M. Multipoint left ventricular pacing with large anatomical separation improves reverse remodeling and response to cardiac resynchronization therapy in responders and non-responders to conventional biventricular pacing. Clin Res Cardiol. (2020) 109:183–93. 10.1007/s00392-019-01499-7 31152199

[13] Crossley GH Biffi M Johnson B Lin A Gras D Hussin A. Performance of a novel left ventricular lead with short bipolar spacing for cardiac resynchronization therapy: primary results of the attain performa quadripolar left ventricular lead study. Heart Rhythm. (2015) 12:751–8. 10.1016/j.hrthm.2014.12.019 25533587

[14] Johar S Luqman N. Early experience with attain stability, an activefixation LV lead: virtues and pitfalls. Pacing Clin Electrophysiol. (2015) 38:297–301. 10.1111/pace.12541 25440812

[15] Erath JW Benz AP Hohnloser SH Vamos M. Clinical outcomes after implantation of quadripolar compared to bipolar left ventricular leads in patients undergoing cardiac resynchronization therapy: a systematic review and meta-analysis. Europace. (2019) 21:1543–9. 10.1093/europace/euz196 31324920

[16] Turakhia MP Cao M Fischer A Nabutovsky Y Sloman LS Dalal N. Reduced mortality associated with quadripolar compared to bipolar left ventricular leads in cardiac resynchronization therapy. JACC Clin Electrophysiol. (2016) 2:426–33. 10.1016/j.jacep.2016.02.007 29759861

[17] Kutyifa V Kosztin A Klein HU Biton Y Nagy VK Solomon SD. Left ventricular lead location and long-term outcomes in cardiac resynchronization therapy patients. JACC Clin Electrophysiol. (2018) 4:1410–20. 10.1016/j.jacep.2018.07.006 30466845

[18] Ypenburg C van Bommel RJ Borleffs CJ Bleeker GB Boersma E Schalij MJ. Long-term prognosis after cardiac resynchronization therapy is related to the extent of left ventricular reverse remodeling at midterm follow-up. J Am Coll Cardiol. (2009) 53:483–90. 10.1016/j.jacc.2008.10.032 19195605

[19] van Everdingen WM Cramer MJ Doevendans PA Meine M. Quadripolar leads in cardiac resynchronization therapy. JACC Clin Electrophysiol. (2015) 1:225–37. 10.1016/j.jacep.2015.07.004 29759311

[20] Verhaert D Grimm RA Puntawangkoon C Wolski K De S Wilkoff BL. Long-term reverse remodeling with cardiac resynchronization therapy: results of extended echocardiographic follow-up. J Am Coll Cardiol. (2010) 55:1788–95. 10.1016/j.jacc.2010.01.022 20413027

[21] Ziacchi M Diemberger I Martignani C Boriani G Biffi M. New left ventricular active fixation lead: the experience of lead extraction. Indian Heart J. (2015) 67 Suppl 3:S97–9. 10.1016/j.ihj.2015.10.379 26995447 PMC4799019

[22] Adler S Kirchhof N Thompson AE Foerster L Marquard KR Hine DS. Two-year extractability of novel left ventricular, active fixation leads in the sheep model. Pacing Clin Electrophysiol. (2017) 40:1291–7. 10.1111/pace.13200 28940232

[23] Chapman M Bates MGD Behar JM Williams I Dewhurst M Monkhouse C. A novel quadripolar active fixation left-ventricular pacing lead for cardiac resynchronization therapy: initial United Kingdom experience. JACC Clin Electrophysiol. (2019) 5:1028–35. 10.1016/j.jacep.2019.05.005 31537331

